# Integrated Model of Metabolism and Autoimmune Response in β-Cell Death and Progression to Type 1 Diabetes

**DOI:** 10.1371/journal.pone.0051909

**Published:** 2012-12-14

**Authors:** Tijana Marinković, Marko Sysi-Aho, Matej Orešič

**Affiliations:** VTT Technical Research Centre of Finland, Espoo, Finland; University of Westminster, United Kingdom

## Abstract

Progression to type 1 diabetes is characterized by complex interactions of environmental, metabolic and immune system factors, involving both degenerative pathways leading to loss of pancreatic β-cells as well as protective pathways. The interplay between the degenerative and protective pathways may hold the key to disease outcomes, but no models have so far captured the two together. Here we propose a mathematical framework, an ordinary differential equation (ODE) model, which integrates metabolism and the immune system in early stages of disease process. We hypothesize that depending on the degree of regulation, autoimmunity may also play a protective role in the initial response to stressors. We assume that β-cell destruction follows two paths of loss: degenerative and autoimmune-induced loss. The two paths are mutually competing, leading to termination of the degenerative loss and further to elimination of the stress signal and the autoimmune response, and ultimately stopping the β-cell loss. The model describes well our observations from clinical and non-clinical studies and allows exploration of how the rate of β-cell loss depends on the amplitude and duration of autoimmune response.

## Introduction

Type 1 diabetes (T1D) is an autoimmune disease characterized by a relatively long symptom-free period that precedes the flair-up of clinical signs of the disease. T1D is caused by progressive loss of insulin-secreting capacity of the β-cells and, finally, by selective death of these cells in the over 1 million islets of Langerhans in the human pancreas. The incidence of T1D among children and adolescents has increased markedly in the Western countries over the recent decades, with the annual incidence continuing to rise at an accelerating pace [1]. Although approximately 80% of subjects with T1D carry defined risk-associated genotypes at the HLA locus, only 3–7% of the carriers of such genetic risk markers develop the disease [2]. Seroconversion to islet autoantibody positivity has been the first detectable signal implicating initiation of autoimmunity and progression towards diabetes [3]. However, although seroconversion to autoantibody positivity precedes the clinical disease by months to several years, the time point at which seroconversion occurs may already be too late for therapeutic approaches aimed at preventing progression to overt diabetes. As long as the initiators of the autoimmune response remain unknown and the mechanisms supporting progression towards β-cell failure are poorly understood, the estimation of disease risk, time of disease presentation in genetically susceptible individuals as well as discovery of effective prevention will remain a challenge.

It is generally accepted that the primary role of the immune system is in protection of the body against foreign pathogens which requires the inhibition of immune response against self. The classical view known as “clonal selection theory” states that potentially pathogenic T cells such as the cells that react specifically to autoantigens should not be present in healthy individuals [4], [5]. However, there is also evidence to the contrary; suggesting autoimmunity may also have a protective role in specific circumstances. For example, it has been shown that autoimmunity may also be a physiological response with a protective role following an insult to the central nervous system [6], [7], [8].

Our metabolomics studies in children who later progressed to T1D (progressors) identified specific metabolic profile preceding the first autoimmune response [9]. Surprisingly, following the autoimmune response this metabolic profile was largely corrected towards the normal levels found in children who did not progress to the disease. This led us to hypothesize that (a) autoimmunity results from physiological adaptation triggered by metabolic stress, and that (b) the subsequent T1D in some individuals is due to defective regulation of the immune response (*e.g.*, its amplitude or timing). Subsequent studies in non-obese diabetic (NOD) mice supported this view as well as identified specific “protective pathways” in mice who seroconverted to insulin autoantibody (IAA) positivity but did not develop autoimmune diabetes [10].

Given the time-dependent complexity of T1D pathogenesis, including interactions across multiple physiological systems, there is a clear case for mathematical modeling in order to capture the complex interplay of the factors involved in the disease process. However, only few attempts to model the disease process have been made. The so-called Copenhagen model [11], [12], [13] focuses on the interplay between activated macrophages, T-helper (Th)-lymphocytes and target cells in the early phases of T1D pathogenesis. According to this model, the mechanism of β-cell destruction as induced by cytokines occurs prior to β-cell destruction by T-lymphocyte mediated mechanisms. This simple model already displayed remarkable complexity and led the authors to conclude that “onset of T1D is due to a collective, dynamical instability, rather than being caused by a single etiological factor” [13]. However, the model does not include protective pathways, or metabolism in general, as factors protecting from or contributing to the disease.

Nevo *et al.* introduced a model of autoimmune response where autoimmunity is viewed as a defense mechanism against tissue-specific degenerative processes [14]. The degenerative process of tissue loss is triggered by the primary insult and it further activates alarm signals which provoke an autoimmune response [15], [16]. The model suggests that anti-self response after being triggered competes with self-perpetuating degenerative tissue loss. Depending on timing and intensity of the autoimmune response, there are three different outcomes of the competition: (a) if the autoimmune response occurs too late or is too weak, it is not able to stop the degenerative process, consequently the self-destruction dominates and tissue degeneration continues; (b) if the autoimmune response fails to stop, regardless of its intensity, the tissue loss continues and ultimately leads to autoimmune disease; (c) autoimmune response can also be protective if it starts early enough and shuts off at the right time, leading to diminishment of the self-degenerative process.

Herein we propose an integrated model of metabolism and autoimmune response to study how they together contribute to progressive β-cell loss and T1D. The model builds on earlier models of β-cell loss [13] and autoimmune response [14] as well as on our longitudinal metabolomics and autoantibody data from children who progressed to T1D in a prospective birth cohort [9] and the data from NOD mouse [10].

## Results and Discussion

Our primary aim is to model the interaction between metabolism and immune system in the early stages of T1D. In summary, β-cell destruction follows two paths of loss: degenerative loss and autoimmune-induced loss. First, the degenerative process of β-cell loss is triggered, *e.g.*, by an infection [17], leading to activation of autoimmune response *via* secretion of a danger signal. The autoimmune response stimulates the autoimmune-induced β-cell loss. The two paths of loss are mutually competing, leading to termination of the degenerative path of loss and further to elimination of the danger signal and the autoimmune response. As a consequence, the autoimmune induced path of loss is terminated as well, stopping the β-cell loss.

A simplified Copenhagen model [13] is assumed for the degenerative path. The process of degenerative loss starts with release of proteins triggered by the environmental factors attacking the β-cells. These proteins are detected by macrophages in the islets of Langerhans, leading to formation of activated macrophages. Activated macrophages release signal molecules (*e.g.*, cytokines) and immunogenic danger signals. Danger (alerting) signals activate the autoimmune response which further triggers the autoimmune-induced process of β-cell loss. Production of cytokines by activated macrophages is on the other hand associated with release of β-cell antigenic proteins. Protective pathway is supposed to be the one that is associated with the seroconversion to autoantibody positivity [9], [10]. Serum lysophosphatidylcholine (lysoPC) is selected to represent such pathway because it is inversely related to insulin autoantibody positivity (IAA+) in NOD progressors and non-progressors to autoimmune diabetes [10], thus reflecting altered association of metabolic and immune system status in disease pathogenesis.

**Figure 1:**
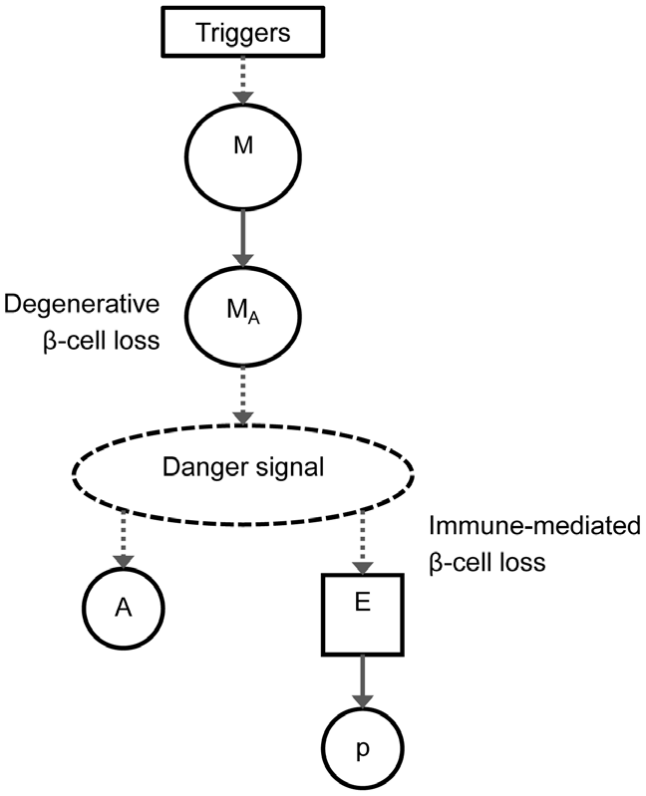
Schematic representation of the model. The parameters and variables of the model are explained in **Table 1**. Proteins released by the environmental triggers attack the β-cells and start the degenerative process of tissue loss. These proteins are detected by macrophages in the islets of Langerhans, leading to formation of activated macrophages. Activated macrophages release signal molecules (*e.g.*, cytokines) and immunogenic danger signals. Danger (alerting) signals activate the autoimmune response which further triggers the autoimmune-induced (“positive”) process of β-cell loss.

Model variables are a combination of variables from Copenhagen model [13] and from protective autoimmunity model [14]: 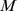 is the amount of macrophages, 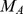 is the amount of activated macrophages, 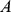 is the amount of beta cell antigenic proteins, as in [13]. 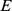 is the strength of autoimmune response and it is introduced in the same sense as autoimmune response variable 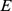 in [14]. 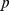 is the concentration of metabolite of protective pathway which is related to autoimmune induced path of loss. This variable has the same meaning as variable 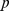 from [14] which represents the population density of cells that undergo the immune mediated (i.e. positive) path of loss. We suggest biological interpretation to this path as a protective metabolic pathway which due to its protectiveness has a positive (defensive) role in beta cell loss. Schematic representation of the model is given in **Figure 1**, with the model variables and parameters shown in **Table 1**. Mathematical model is described in detail in **Materials and Methods** section.

**Table 1:** Description of the model variables and parameters.

**Variables**	
M	Amount of macrophages
M_A_	Amount of activated macrophages
A	Amount of β-cells antigenic proteins
E	Autoimmune response
P	Protective pathway, metabolite
**Parameters: Left branch (degenerative path of β-cell** **loss, as in Copenhagen model)**	
*a*	inflow rate of macrophages
*b*	inflow rate induced by activated macrophages
*c*	Decay rate of macrophages
*g*	Rate of activation of macrophages
*k*	Decay rate of activated macrophages
**Parameters: right branch (immune-mediated path of β-cell** **loss, i.e., protective pathway)**	
*k_x_*	Reaction rate constant for the enzymatic reaction of the protective pathway (xp)
*k_e_*	Decay rate of positive path
**Parameters: connection between left and right branches**	
*l*	Rate of creation of β-cell antigenic proteins
*m*	Decay rate of β-cell antigenic proteins
S_S_⋅*l*⋅*g*	Amplitude parameter of autoimmune response
*S_E_*	Decay of autoimmune response
*E*_1_	Threshold value for activation of autoimmune response
*E*_2_	Threshold value for shut-off of autoimmune response

**Figure 2:**
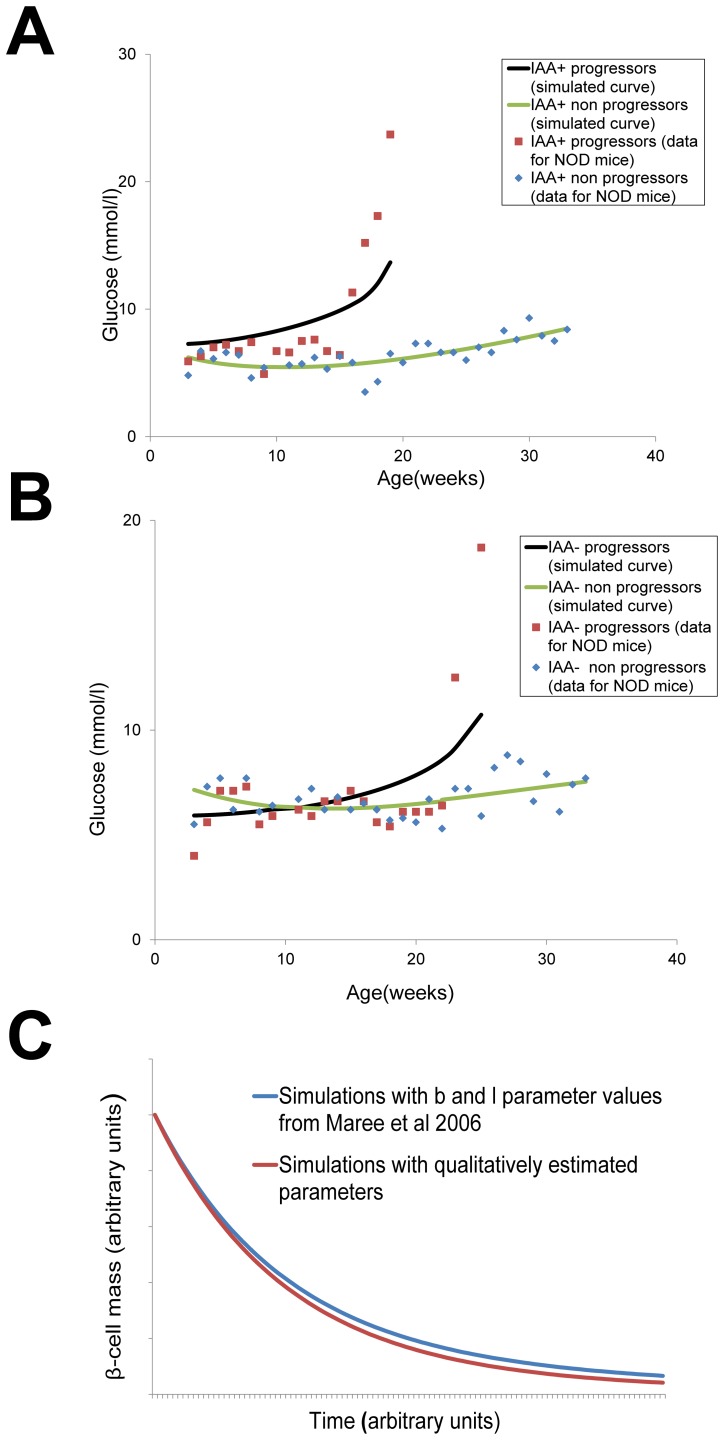
Model predictions of age-dependent glucose profiles in disease progressors and non-progressors and model validation based on prediction of β-cell mass. (A) Model prediction of glucose levels in autoantibody positive cases. (B) Model prediction of glucose levels in autoantibody positive cases. The profiles are fitted to glucose measurements in NOD mice from [10] which are shown in the same figure. The goodness-of-fit *R^2^* values are 0.83 (IAA+ progessors), 0.55 (IAA+ non-progressors), 0.59 (IAA- progressors) and 0.18 (IAA- non-progressors). Fitting was performed using the fminsearch function in Matlab (Mathworks, Inc., Natick, MA). Because the number of experimental data points is small (17 data points for IAA+ progressors, 30 data points for IAA+ non progressors, 21 data points for IAA- progressors and 31 data points for IAA- non progressors), we fit the trends of data rather than their exact behaviour. (C) Prediction of β-cell mass: model performances with a set of qualitatively estimated parameters for IAA+ progressors case and with 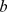 and 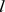 parameter value taken from [19].

Since the experimental longitudinal data on β-cell mass are not available, we use experimental longitudinal data of glucose concentrations from NOD mice [10] and apply the model by [18] in order to estimate β-cell mass and select the model parameters (**Figures 2A and 2B**). The mice are divided into four groups: (a) progressors to autoimmune diabetes who were IAA+ when measured at age of 8 weeks, (b) non-progressors who were IAA+ at 8 weeks of age (“protected” mice), (c) progressors who were IAA- at 8 weeks of age, and (d) non-progressors who were IAA- at 8 weeks of age. As a verification of our qualitatively estimated parameters model simulations are performed with parameters values of 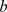 (recruitment rate of macrophages by activated i.e. inflow rate induced by activated macrophages) and 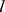 (beta cell apoptosis induced by activated macrophages i.e. rate of creation of beta cell antigenic proteins) for NOD mice which were estimated based on experiments and literature [19]. Simulated profiles obtained with our qualitatively estimated set of parameters for the case of IAA+ progressors fit well the simulations reproduced with two estimated parameters from [19]. A comparison of beta cell profiles is shown in **Figure 2C**.

**Figure 3:**
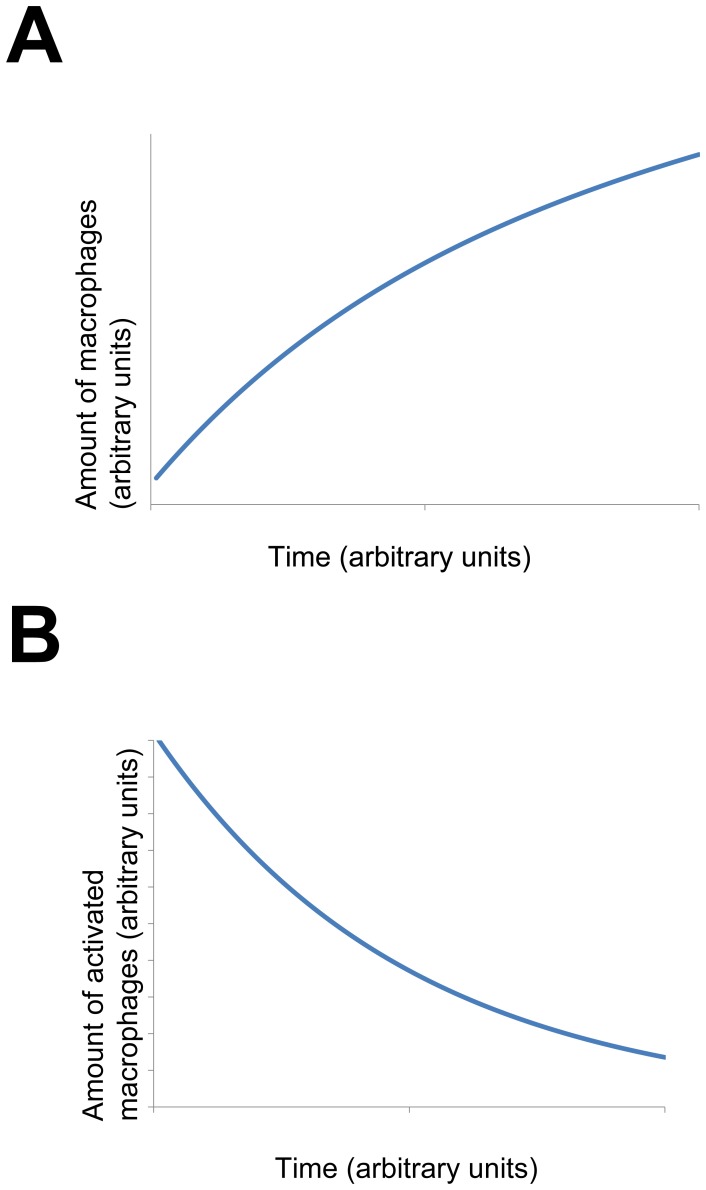
Simulated amounts of macrophages and of activated macrophages. The amount of macrophages increases with time and the amount of activated macrophages decreases in time (in agreement with the Copenhagen model [13]).

The differential equations determine the values of the simulation results that depend on altogether nine parameters. Among these, 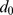, 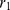 and 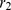 affect the shape of the simulated glucose curve (see equations 6.1–6.3 in **Materials and Methods**). Values for these parameters were set by fitting the model outcomes to the experimental data from [10] using multidimensional unconstrained nonlinear minimization based on Nelder-Mead method [20]. Since the number of experimental points in available datasets is quite small (17 points for IAA+ progressors, 30 points for IAA- progressors, 21 points for IAA+ non-progressors and 31 points for IAA- non-progressors) one cannot properly draw conclusions about the exact behavior of data (i.e. about the shapes of the data curves). We rather limit ourselves to interpret and to fit the trends of data: how fast the concentration of glucose tends to increase depending on conditions of IAA positivity and progressivity or non to the disease. Small oscillations in non progressors data are neglected and the trend in these data sets are interpreted as slowly increasing (almost constant). Fitting with such a small but only available data set is not precise enough which can be also seen from the goodness-of-fit 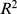 values (e.g. IAA- non-progressors 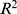 is quite low). According to our model the amount of macrophages increases while the amount of activated macrophages decreases with time (**Figure 3**). This is in agreement with the Copenhagen model [13], which explains that after an initial time during which the number of macrophages drops down because of antigen uptake, it increases later on because of activation-induced inflow and deactivation. The number of activated macrophages according to Copenhagen model after an initial period during which it grows because antigens are taken up, it declines later on when the antigens have been swept away.

**Figure 4:**
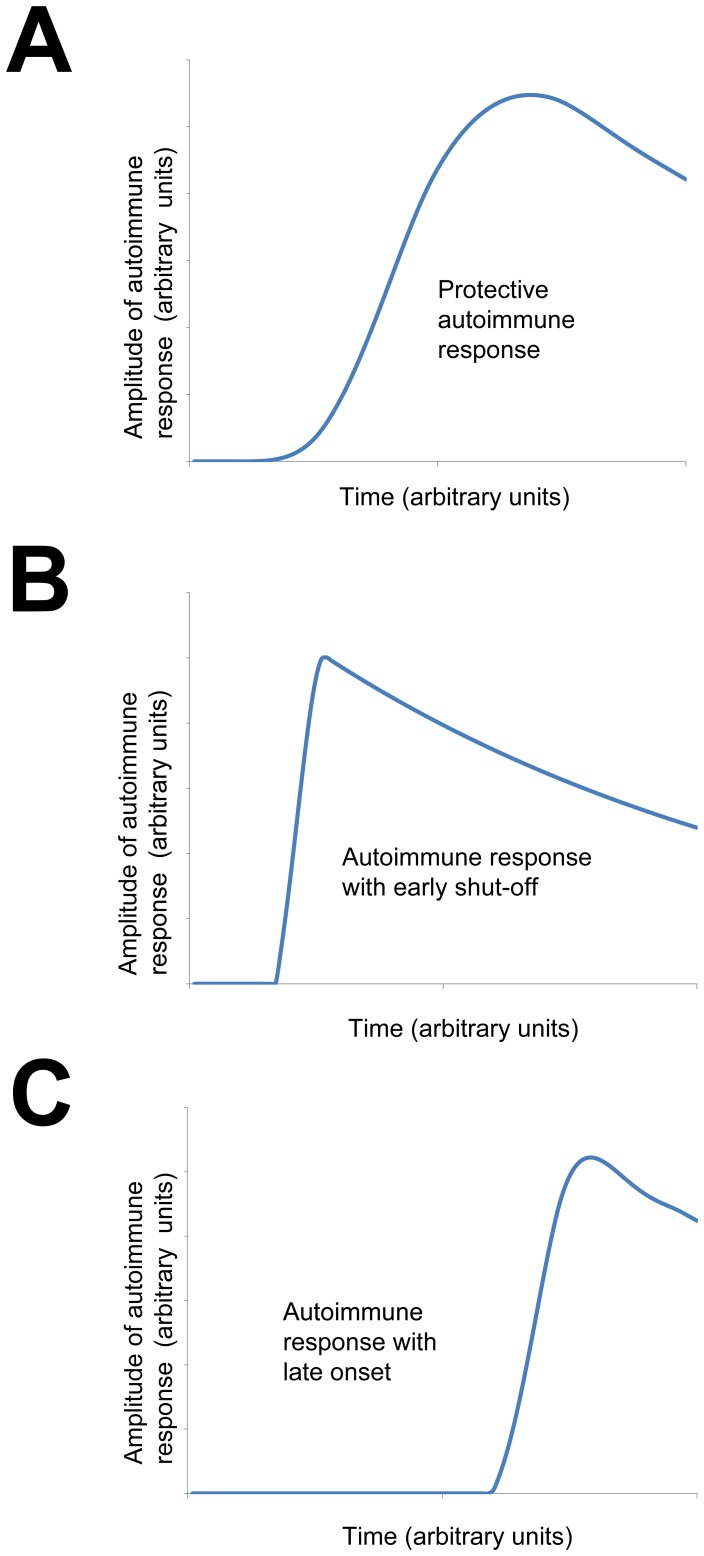
Different time courses of autoimmune response. Simulated (**A**) protective autoimmunity, (**B**) autoimmunity with an early shut-off and (**C**) delayed-onset autoimmunity. Different profiles are obtained by setting different values of 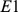 and 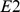 parameters (equation 2).

As part of qualitative evaluation of the model, intensity profiles of autoimmune response were compared to the ones reported in [14]. Up to the authors knowledge these simulated profiles of autoimmunity are the only ones found in literature and there are no experimental profiles available. The protective autoimmune response simulated by our model appears to agree with the simulated protective autoimmune response reported in [14]. Comparison between simulated autoimmune responses for different values of parameters 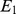 and 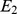 (equation 2 in **Materials and Methods**) is shown in **Figure 4**. Autoimmune response simulated by setting parameter 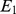 to be higher than the one in protective autoimmunity can be interpreted as autoimmune response with premature shut-off (**Figure 4B**). Autoimmune response simulated by setting parameter 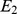 to be lower than the one in protective autoimmunity corresponds to delayed-onset autoimmunity (**Figure 4C**).

**Figure 5:**
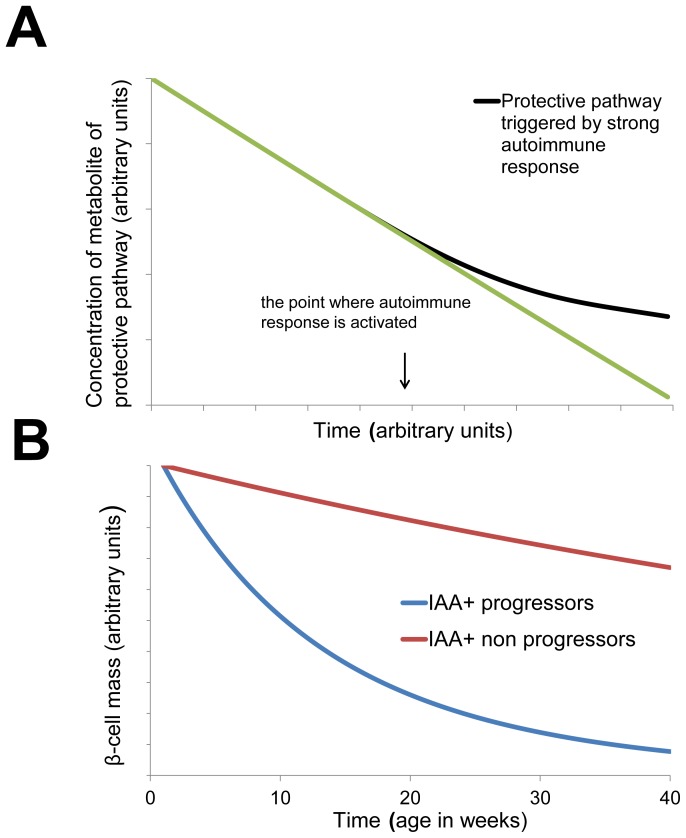
Protective pathway and progression to autoimmune diabetes. (**A**) Interaction between protective metabolic pathway and autoimmune response. Autoimmune response with stronger intensity leads to higher concentration of metabolite of protective pathway (effective protective pathway) as compared to the autoimmune response with weak intensity (defective protective pathway). (**B**) Prediction of β-cell mass in disease progressors and in protected non-progressors. The simulation is based on experimental data for NOD mice. Progressors are IAA+ at 8 weeks of age and non-progressors are IAA+ at 8 weeks of age [10]. β-cell loss is slower in the presence of effective protective pathway.

Next, we studied time profiles of the protective pathway and β-cell loss (**Figure 5**). The influence of intensity of autoimmune response on activation of protective pathway is shown in **Figure 5A**. Once autoimmune response is triggered, protective pathway behaves differently depending on the intensity of autoimmune response. Autoimmune response which is strong enough leads to creation of effective protective pathway and weak autoimmune response provokes defective protective pathway. When autoimmune response is stronger, the concentration of metabolite of protective pathway is higher. This is in agreement with the changes of serum lysoPC in NOD mice in the context of IAA positivity and progression to autoimmune diabetes [10], where it has been observed that IAA+ positive mice with high lysoPC were protected from autoimmune diabetes. When comparing autoimmune progressors (defective protective pathway) and non-progressors (effective protective pathway), the model predicted that rate of β-cell loss is markedly slowed in the presence of protective pathway (**Figure 5B**). It is assumed that all the underlying metabolic mechanisms are implicitly included in the protective pathway so as the role of T cells in the pathogenesis of T1D.

While inclusion of the protective pathway in the model of β-cell loss shows promise for the study of mechanisms behind protection from T1D, at present it is generalized by taking one enzymatic reaction for the whole protective pathway. This is clearly an over-simplification. In the future, the protective path equations need to be developed for a realistic pathway and to take into account explicitly the role of T cells in progression to T1D. For example, in our earlier study of NOD mice [10] we identified specific inflammatory pathways in isolated pancreatic islets which were up-regulated in IAA+ non-progressors. Among those, product of IL-4 pathway is in fact known to prevent diabetes in NOD mouse [21]. The serum lysoPC used in the present study is an inflammatory marker [22] and its up-regulation in IAA+ non-progressors may therefore reflect the activation of protective inflammatory pathways at specific stage following the autoimmune response. As another candidate protective pathway, islet pathways related to mitochondrial function such as TCA cycle, branched chain amino acid catabolism, beta oxidation and oxidative phosphorylation, were down-regulated in IAA+ non-progressors. These pathways lead to reduced production of reactive oxygen species (ROS) [10]. Since ROS generated by mitochondria plays an important role in the release of pro-apoptotic proteins which can trigger apoptosis [23], decreased ROS production could thus be linked to prevention of loss of β-cell functionality in IAA+ non-progressors. Incorporation of more detailed pathways such as those described above into the specific parts of the model may identify specific molecular targets behind the control of the protective pathway.

In summary, herein we presented the first model of β-cell loss in progression to T1D which also includes the protective pathway; with the model parameters fitted using the experimental data from NOD mouse. The model predicts that the autoimmune response, if properly tuned, can also have a protective role, leading to the reduced rate of β-cell loss. At present, the model is qualitative but it already allows exploration of how the rate of β-cell loss depends on the amplitude and duration of autoimmune response, which in turns depends on the activation of protective pathways. As such, it is also a useful conceptual tool for the study of how immune system interacts with other physiological systems in progression to autoimmune disease.

## Materials and Methods

### Mathematical Model

In the degenerative path of loss, () equations from Copenhagen model [13] are applied. These equations describe changes in numbers of macrophages (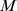), activated macrophages (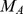) and β-cell antigenic proteins (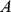), where number of β-cell antigenic proteins corresponds to the amount of β-cells. Since the death of β-cells leads to release of proteins, it can be assumed that the rate of death of β-cells is proportional to the amount of activated macrophages [13]. There is therefore no need to explicitly include the β-cell population in the equations because release of proteins is directly related to the number of activated macrophages. Three ordinary differential equations for this path are:
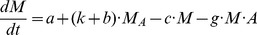
(1.1)

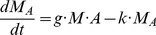
(1.2)

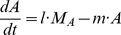
(1.3)


Parameters 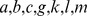
are rate parameters of different mechanisms included in progressive loss of β-cells. Their description is given in **Table 1** and it follows the ones given in [13].

**Figure 6:**
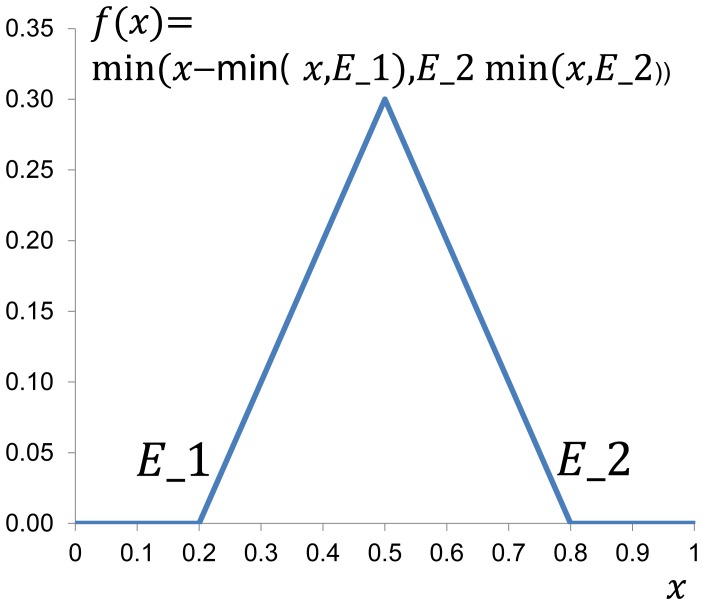
Schematic presentation of function used in autoimmune response equation. 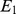
 is the threshold for autoimmune response activation and 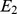
is the shut-off value.

Equation for autoimmune response is inspired by the equation of the mean level of immune stimulation [14] which introduces a sigmoid function of the spatially accumulated alerting signal with a treshold value below which the induction of immune stimulation is negligible. Variables in our model are assumed as functions of time only. Danger (alerting) signal is not explicitly included but it is assumed that activated macrophages induce autoimmune response *via* danger signals. Therefore instead of sigmoid function of spatially accumulated danger signal we introduce a simplified function of activated macrophages 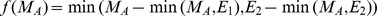 to the equation for autoimmune response. This function accounts for two limit values 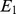 (activation value) and 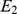 (shut-off value). The function is chose so that below 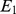 autoimmune response is not activated (analogously to the threshold value of the sigmoid function from [14]) and above 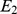 autoimmune response is closed (**Figure 6**). This equation is given as
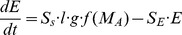
(2)


Because of the assumption that autoimmune response is created from activated macrophages and the hypothesis that rate of death of β-cell depends on the strength of autoimmune response, the amplitude of the autoimmune response is taken to be proportional to the product of parameters 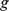 (rate of activation of macrophages) and 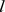
(rate of creation of β-cell antigenic proteins) in the form 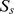. 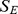 is decay of autoimmune response. Danger signal is not introduced as a separate variable but it is implicitly included in the equation which describes autoimmune response.

Protective pathway is simplified by assuming only one enzymatic reaction and one relevant metabolite for the whole metabolic pathway. The corresponding equation for the protective pathway is motivated by differential equations for the concentrations of metabolites given in [24] and the equation of the rate of transition of positive path [14]. This equation therefore takes the following form
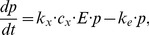
(3)where 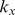 is the reaction rate constant of the enzymatic reaction of the protective pathway,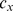 is the concentration of the initial metabolite in the reaction and 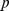 is the concentration of the observed metabolite which represents the protective pathway and 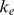 is the decay rate. The influence of autoimmune response immune-mediated path of loss is expressed through multiplication with 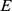.Immune-mediated path is triggered after activation of autoimmune response i.e. above activation threshold value 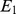. Protective metabolic pathway is represented by serum lysoPC metabolite, which is upregulated in IAA+ non-progressor NOD mice [10].

Complete system of equations for the model is resumed below:
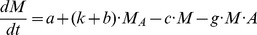
(4.1)

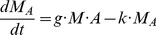
(4.2)

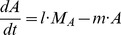
(4.3)

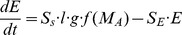
(4.4)

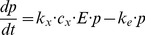
(4.5)


Description of all the parameters and variables of the system of five ordinary differential equations is given in **Table 1**.

### Connecting β-cell Mass with Glucose Levels

The only available experimental data associated with β-cell mass are glucose concentration data. In order to connect β-cell mass with glucose levels an additional part of the model is developed that is based on combination between Copenhagen model equations (equations 4.1–4.3) and β-cell mass, insulin and glucose kinetics model equations [18]:
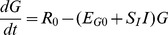
(5.1)

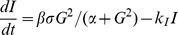
(5.2)

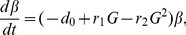
(5.3)where 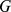 is the glucose concentration, 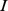 is the insulin concentration and 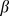 is the mass of β-cells. Short description of variables and parameters used in equations 5 according to [18] can be found in **Table 2**. According to [18] glucose and insulin dynamics are fast relative to 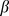
cell mass dynamics. Therefore the 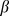 cell mass, insulin and glucose model can be decomposed into fast (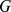
_,
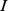
_) and slow (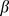 cell mass) subsystems. To study the slow subsystem it is assumed that the fast subsystem is at steady state. The right side of equation 4.3., where parameter A represents β-cell mass, is equivalent with the right side of equation 5.3, resulting in expressing variable 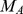 (activated macrophages) in terms of variable 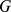
_,_ equation (6.2). When evolving equation (6.2) the steady states of glucose and insulin are assumed according to the assumption made in [18], as explained above. Consequently, resulting system of three ordinary differential equations is obtained:
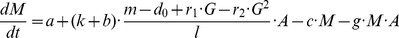
(6.1)

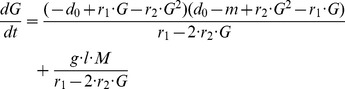
(6.2)

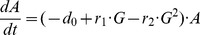
(6.3)


**Table 2:** Description of the variables and parameters from the β-cell model.

Name	Description
*G*	Glucose concentration
*I*	Insulin concentration
*β*	B-cell mass
*R*_0_	Net rate of production at zero glucose
*E_G_*_0_	Total glucose effectiveness at zero insulin
*S_I_*	Total insulin sensitivity
*σ*	Maximal rate of insulin secretion
_G/(*α* + G_^2^_)_	Hill function with coefficient 2 describing sigmoid ranging from 0 to 1 which reaches half it maximum at G = α^1/2^)
*k_I_*	Clearance coefficient of insulin
*d*_0_	Death rate at zero glucose
*r*_1_	Rate constant
r_2_	Rate constant

For more detail about the original model of coupled β-cell mass, insulin and glucose dynamics one should see [18]. We find such model suitable for needs of our model because it gives a way to obtain shape of β-cell mass from the profile of glucose which is reproduced from experimental data.

The whole model thus consists of two parts: the “main” part expressed through equations 4.1–4.5 and the “auxiliary” part expressed through equations 6.1–6.3. There are 8 ordinary differential equations in total and 6 variables. Variables 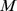 and 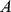 are connecting the two parts of the model.
